# Insulin and osteocalcin: further evidence for a mutual cross-talk

**DOI:** 10.1007/s12020-017-1396-0

**Published:** 2017-09-02

**Authors:** Francesco L. Bilotta, Biagio Arcidiacono, Sebastiano Messineo, Marta Greco, Eusebio Chiefari, Domenico Britti, Tomoko Nakanishi, Daniela P. Foti, Antonio Brunetti

**Affiliations:** 1 Department of Health Sciences, University “Magna Græcia” of Catanzaro, Viale Europa (Località Germaneto), 88100 Catanzaro, Italy; 2 Laboratory of Molecular Genetics, The Institute of Medical Science, University of Tokyo, 108-8639 Tokyo, Japan

**Keywords:** Bone, Osteocalcin, Insulin action, Type 2 diabetes

## Abstract

**Purpose:**

In the last few years, bone has been recognized as an endocrine organ that modulates glucose metabolism by secretion of osteocalcin, an osteoblast-specific hormone, that influences fat deposition and blood sugar levels. To date, however, very few in vitro models have been developed to investigate, at the molecular levels, the relationship between glucose, insulin and osteocalcin. This study aims at covering this gap.

**Methods:**

We studied osteogenic differentiation, *osteocalcin* gene expression, and osteblast-mediated insulin secretion, using cultured MG-63 human osteoblast-like cells that underwent glucotoxicity and insulin resistance. In addition, we investigated whether a correlation existed between hyperglycemia and/or insulin resistance and total osteocalcin serum concentrations in patients.

**Results:**

While insulin and low glucose increased *osteocalcin* gene expression, disruption of insulin signaling in MG-63 osteoblasts and high glucose concentration in cell culture medium decreased *osteocalcin* gene transcription and reduced osteogenic differentiation. Concomitantly, insulin secretion was significantly impaired in rat INS-1 β-cells treated with conditioned medium from insulin resistant MG-63 cells or cells exposed to high glucose concentrations. Also, chronic hyperglycemia, but not insulin resistance, inversely correlated with circulating osteocalcin levels in patients.

**Conclusion:**

Our results further support the existence of an endocrine axis between bone, where osteocalcin is produced, and pancreatic β-cells, and add new insights into the molecular details of this relationship. These findings may contribute to the understanding of osteocalcin regulation and its role in metabolism.

## Introduction

The regulation of glucose metabolism in mammals is a complex physiological process based on the interplay of a variety of hormones and other extracellular signals acting on specific target tissues. Among the main hormones involved in this process, insulin plays a major role in promoting glucose uptake in muscle and adipose tissue, while it suppresses gluconeogenesis in the liver [1], thereby reducing postprandial glucose levels in the blood. Because of the fundamental importance of insulin within this context, the insulin receptor signaling pathway has been studied intensively through the past decades, with the identification, in recent years, of novel non-canonical insulin target tissues that appear to play important roles in whole body glucose homeostasis. In line with this finding, the existence of an insulin receptor signaling pathway has been defined recently in the bone, specifically in osteoblasts [2], leading to the conclusion that bone can be a target of insulin action and a major regulator of energy metabolism by favoring β-cell insulin secretion and peripheral insulin sensitivity through an intricate array of hormones and other factors acting locally and systemically [3, 4].

In particular, a key role in the regulation of glucose metabolism, at this level, is attributed to osteoblasts [5], which produce the osteoblast-specific protein, osteocalcin, the most abundant non-collagenous protein in the bone. Before being secreted into blood, osteocalcin is carboxylated on three glutamine residues (Bone Gla Protein) [6] through vitamin K-dependent mechanisms. The carboxylation of osteocalcin confers high affinity for bone matrix, whereas decarboxylation generates the formation and activation of the hormonal form of osteocalcin [7]. The evidence that osteocalcin may have an extraskeletal metabolic role beyond its effect on bone is supported by data from mice lacking osteocalcin, in which an increase in visceral fat followed by hyperglycemia and hypoinsulinemia was observed, together with a reduced β-cell mass and insulin content [8]. Besides promoting insulin secretion, osteocalcin increases peripheral insulin sensitivity [9, 10]. Interestingly, in mice lacking either one copy of the *insulin receptor* gene in osteoblasts, or the *osteocalcin* gene, high fat diet (HFD) induced a more severe form of insulin resistance than wild-type mice did [11], whereas selective overexpression of the *insulin receptor* in osteoblasts protected from HFD-induced insulin resistance.

Based on the experimental findings so far available, the existence of a positive loop between osteocalcin and insulin is suggested, by which osteocalcin stimulates insulin secretion, while the release of osteocalcin is enhanced by insulin. In support of this view, patients with insulin resistance and abnormalities in glucose metabolism have low circulating osteocalcin levels, and may have consequences in their bone structure and function (e.g., higher risk of fractures) [12], thereby further strengthen the link between skeletal and glucose metabolism.

In the present study, we confirm and extend previous observations linking osteocalcin and glucose metabolism. Using in vitro models of insulin resistance, we provide data that can help to better understand the role of osteocalcin in the pathophysiology of insulin deficiency and the events leading to insulin-resistant states and metabolic disorders.

## Materials and methods

### Cells

MG-63 (ATCC CRL-1427) human osteosarcoma cells and the human embryonic kidney HEK-293 cell line [13] were cultured in Dulbecco's Modified Eagle's medium (DMEM) (Invitrogen) supplemented with 10% fetal bovine serum (FBS), 2 mM glutamine, penicillin (100 U/mL), and streptomycin (100 µg/mL) in a humidified 5% CO_2_ atmosphere at 37 °C. INS-1 rat insulinoma cells [13] were cultured in RPMI-1640 medium (Sigma-Aldrich) supplemented with 10% FBS, 2 mM glutamine, penicillin (100 U/mL), streptomycin (100 μg/mL), 50 μM beta-mercaptoethanol, and 100 mM HEPES buffer (Sigma-Aldrich). The culture medium was replaced every 3 days.

### Osteogenic differentiation

MG-63 cells were seeded at approximately 3000 cells/cm^2^ in 24-well culture plates and maintained in an incubator at 37 °C and 5% CO_2_. After 48 h, the standard medium was replaced with osteogenic medium, consisting of DMEM with 10% FBS, 50 μg/mL 2P-ascorbic acid, 100 nM dexamethasone, 10 mM β-Glycerophosphate disodium salthydrate, 2 mM L-glutamine, 100 U/ml penicillin, 100 μg/mL streptomycin. The culture medium was changed every 3–4 days until 14 days of culture.

### Induction of insulin resistance

Palmitic acid (Sigma-Aldrich) was first dissolved in ethanol at a concentration of 200 mM and then diluted (1:20) with phosphate-buffered saline (PBS) 1X, plus 10% fatty acid free BSA (Sigma-Aldrich) at 65 °C for 15 min. Palmitate was added into cell culture medium at 200 μM for 2 days to induce insulin resistance.

### Treatment with glucose and insulin

Low, standard or high glucose concentrations (2, 5, and 25 mM, respectively) were added to glucose-free culture medium starting from 24 h after seeding (on day 1) and after each medium change during the time course experiments. To examine insulin signaling, cells were treated with 10 nM Insulin (Sigma-Aldrich) [11].

### Culture-conditioned medium from MG-63 cells and insulin secretion

Insulin resistant and non-insulin resistant MG-63 cells were cultured and differentiated for 14 days in osteogenic medium. Subsequently, the culture conditioned medium was collected and centrifuged for 10 min at 1500 ×* g* (to remove cells and cell debris in the solution) and then filtered and stored at −80 °C. INS-1 cells were grown in RPMI-1640 at 80% confluence, and starved overnight in Hank’s Balanced Salt Solution (HBSS) buffer, supplemented with standard 5 mM glucose. The following morning, cells were switched to fresh HBSS buffer and insulin secretion was measured at 3 and 15 mM glucose, with and without the addition of conditioned medium from insulin resistant and non-insulin resistant MG-63 cells [14].

### Reporter assay

For luciferase reporter assays, 2 μg of pOC-Luc (*luciferase* gene with the human *osteocalcin* promoter) or pΔOC-Luc (*luciferase* gene without the *osteocalcin* promoter) [15], were transiently transfected into MG-63 and HEK-293 cells, using the LipofectAMINE 2000 reagent (Thermofisher), and luciferase activity was assayed 48 h later, using the dual-luciferase reporter assay system (Promega) [16]. The effects of different concentrations of glucose on the *osteocalcin* promoter were tested after 48 h of stimulation.

### Detection and quantification of mineralization

Esperiments were performed as previously described [17]. MG-63 monolayers in 24-well plates (2 cm^2^/well) were washed with PBS and fixed in 10% (v/v) formaldehyde. The monolayers were then washed with PBS prior to addition of 1 mL of 40 mM Alizarin Red S (ARS, Sigma-Aldrich) per well. The plates were incubated for 20 min. After aspiration of the unincorporated dye, the wells were washed five times with PBS while shaking for 5 min. The plates were then stored at −20 °C prior to dye extraction. Stained monolayers were visualized by phase microscopy using an inverted microscope (Leica). For quantification of staining, 500 µL 10% (v/v) acetic acid was added to each well, and plates incubated at room temperature for 30 min with shaking. The monolayer was then scraped from the plate and transferred with 10% (v/v) acetic acid to a 1.5-mL microcentrifuge tube. The slurry was heated to exactly 85 °C for 10 min, and transferred to ice for 5 min. The slurry was then centrifuged at 20.000 g for 15 min and 500 µL of the supernatant was removed to a new 1.5-mL microcentrifuge tube. Then, 150 µL of 10% (v/v) ammonium hydroxide was added to neutralize the acid. Aliquots (100 µL) of the supernatant were read in triplicate at 405 nm in 96-well format using opaque-walled, transparent-bottomed plates.

### Protein extract and western blot

Cells were washed with PBS, harvested and the cell pellet resuspended in radioimmunoprecipitation assay buffer (50 mM Tris-HCl, pH 7.4; 1% NP-40; 0.5% Na-deoxycholate; 0.1% SDS; 1 mM EGTA; 1 mM PMSF; 150 mM NaCl; 1 mM Na-orthovanadate; 10 mM NaF; anti-proteases). Final protein concentrations in the extracts were determined using the colorimetric assay of Bradford. Total protein extracts were resolved on sodium dodecyl sulfate polyacrylamide gel electrophoresis and electrotrasferred onto a 0.2 μm PVDF membrane (Merck-Millipore) [18]. The antibodies used for these studies were: anti-phospho-AKT Ser473 (Cell Signaling), anti-AKT (Santa Cruz Biotechnology), anti-phospho IRS-1 Tyr612 (Merck-Millipore) and anti-IRS-1 (Abcam), anti-OCN (Merck-Millipore), anti-β-tubulin (Cell Signaling). The resulting immunocomplexes were visualized by enhanced chemiluminescence.

### RNA extraction and qRT-PCR analysis

Total cellular RNA was extracted with Trizol (Invitrogen) [19] and subjected to DNase treatment (Ambion). Amounts were normalized against ribosomal RNA in each sample. cDNAs were synthesized from 2 µg of total RNA using the RETROscript first strand synthesis kit (Ambion). A real-time thermocycler (Eppendorf Mastercycler ep realplex ES) was used to perform qRT-PCR. SYBR Green fluorescence was measured, and relative quantification was made against the *RPS9* cDNA used as an internal standard. Gene-specific primers for qRT-PCR (rat *InsI* for GACCCGCAAGTGCCACAA, rev TCCACAAGCCACGCTTCTG; human *OCN* for TGACGAGTTGGCTGACCA, rev AGGGTGCCTGGAGAGGAG) were designed according to sequences from the GenBank database.

### Insulin assay

Insulin levels were measured in cell culture medium using the rat insulin ELISA kit produced by Mercodia Co., Sweden, and an EIA plate reader with a 450 nm filter (Statfax 2100, USA) cells [14].

### Clinical data

A total of 64 consecutive unrelated subjects with and without type 2 diabetes mellitus, attending the Operative Unit of Endocrinology (University “Magna Græcia”, Catanzaro), were recruited in the study (Table 1). In affected individuals, diabetes was diagnosed according to the American Diabetes Association criteria based on fasting glucose levels (126 mg/dL) [20]. None of them were taking any hypoglycemic medications, and diabetes was controlled by diet only, both in new patients and in diabetic patients with glycated hemoglobin (HbA1c) below 7% (53.0 mmol/mol) at the previous visit. Reasons for exclusion included osteoporosis (*T* score ≤ –2.5) and conditions/drug treatments known to influence bone homeostasis. In non-diabetic subjects, bone metabolism-related diseases such as hypothyroidism, hyperthyroidism, primary hyperparathyroidism and osteoporosis were excluded on clinical ground, together with the exclusion of drugs affecting bone mineral metabolism. In all subjects, blood was withdrawn for the contextual determination of serum glucose, insulin and HbA1c, while an aliquot was stored at −80 °C for the subsequent analysis of total osteocalcin. Using the homeostatic model assessment for insulin resistance (HOMA-IR), patients were classified as insulin sensitive (HOMA-IR < 1.6) or insulin resistant (HOMA-IR > 2.5). Blood glucose, insulin and HbA1c were measured by standard techniques with the instruments Cobas 6000 (Roche), Advia Centaur (Siemens) and HA8160 (Menarini), respectively. Imprecision data were as follows: serum glucose average intra-assay CV = 1.8%, inter-assay CV = 2.1%; serum insulin average intra-assay CV = 4.9%, inter-assay CV = 5.2%; HbA1c average intra-assay CV = 0.9%, inter-assay CV = 1.4%. HOMA-IR was calculated by the formula: glucose (mg/dL) x insulin (mU/L)/405. Total serum osteocalcin was measured by a one-step chemiluminescent immunoassay (Liaison, DiaSorin). Serum osteocalcin average intra-assay CV = 4.9%, inter-assay CV = 6.7%. The study was approved by the local ethics committee, Regione Calabria Comitato Etico Sezione Area Centro (protocol registry n. 116 of May 14, 2015). Written informed consent was obtained from all individual participants included in the study.

**Table 1:** Demographic, anthropometric, clinical and biochemical features of selected subjects

Features	T2D *N* = 22	Control *N* = 42	*P*
Ethnicity	Caucasian	Caucasian	–
Female (n,)	7 (60.0)	23 (50.0)	0.114
Age (yr)	67 (56–73)	59 (50–70)	0.092
BMI (Kg/m^2^)	27.0 (24.5–29.3)	25.5 (24.3–29.8)	0.415
FPG (mg/dL)	158 (117–184)	94 (86–101)	<0.001
HbA1c (%)	7.9 (7.6–8.8)	5.6 (5.4–5.8)	<0.001
HbA1c (mmol/mol)	62.8 (59.6–72.7)	37.7 (35.5–39.9)	<0.001
HOMA-IR	6.9 (3.7–18.6)	3.9 (1.2–5.4)	<0.001
Insulin (mU/L)	18 (11–44)	15.5 (6–23)	0.038

Data are medians (IQR), or *n* (%). Non-parametric Mann-Whitney test was used for distribution comparisons of quantitative variables. The two-tailed Fisher exact test was used for proportion comparisons between groups *BMI* body mass index, *FPG* fasting plasma glucose, *HOMA-IR* homeostatic model assessment method of insulin resistance

Significance level *P* < 0.05

### Statistical analysis

Each quantitative trait was analyzed for distribution using the Shapiro–Wilk normality test and, when required, it was log-transformed. Continuous variables are expressed as median and interquartile range (IQR), and categorical data as number and percentage of the total. Comparison among two groups was performed with the non-parametric Mann–Whitney for continuous variables between two groups and with the 2-tailed Fisher exact test for proportions. Linear regression analysis was employed to examine the quantitative variables that were independently associated with osteocalcin levels, adding appropriate covariates. Statistical significance was inferred at a 2-tailed *P* value < 0.05. Analyses were performed using the SPSS 20.0 software (SPSS Inc., Chicago, IL, USA).

## Results

### Palmitate causes insulin resistance in MG-63 cells

**Fig. 1:**
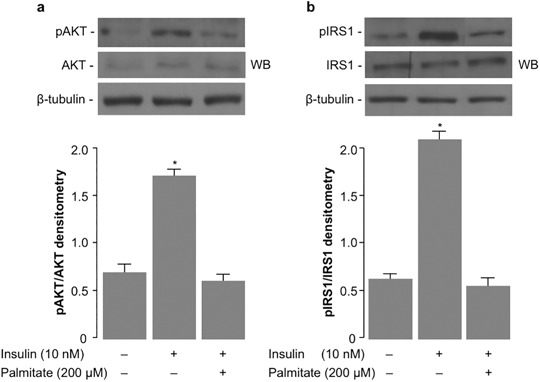
Palmitate reduces insulin signaling in MG-63 osteoblast-like cells. **a** Representative western blots (WB) of phosphorylated (pAKT) and unphosphorylated AKT in MG-63 control cells (–), and in cells stimulated with insulin (+) for 15 min, either in the absence or presence of 200 µM palmitate for 48 h. **b** Representative WBs of phosphorylated (pIRS-1) and unphosphorylated IRS-1 in MG-63 cells treated as in **a**. β-tubulin, control of protein loading. Densitometric analyses of three independent blots (mean ± SEM) from three independent experiments are shown in each condition. *P < 0.05 vs. insulin-untreated control cells

As an insulin-sensitive cell type [21], MG-63 cells were chosen as an appropriate in vitro osteoblastic-like model to study the role of insulin in osteoblasts. Palmitate, a typical saturated free fatty acid was used to induce insulin resistance in these cells, thereby reducing insulin signaling. As shown in Fig. 1a, treatment of MG-63 cells with 10 nM insulin induced AKT phosphorylation, whereas this induction was prevented in cells pre-treated with 200 µM palmitate. Likewise, in parallel experiments, while insulin induced IRS-1 phosphorylation in untreated insulin sensitive MG-63 cells, insulin stimulation of IRS-1 phosphorylation was impaired in the presence of palmitate (Fig. 1b).

### Insulin resistance and high glucose reduce *osteocalcin* gene expression in MG-63 cells

**Fig. 2:**
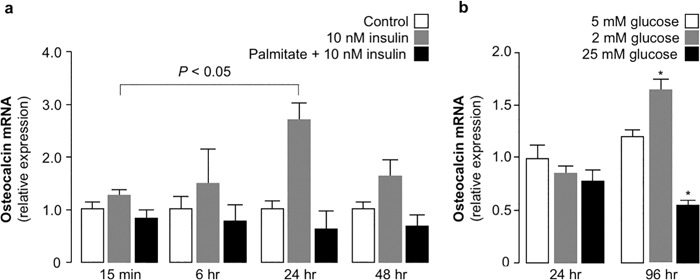
Insulin resistance and high glucose prevent *osteocalcin* gene expression in MG-63 cells. **a** qRT-PCR analysis of the expression of *osteocalcin* gene in untreated (Control), or insulin-treated MG-63 cells for up to 48 h, with and without pre-treatment with 200 µM palmitate. In all qRT-PCR experiments, data shown represent means ± SEM of three independent experiments, each in triplicate. **b** qRT-PCR analysis of the expression of *osteocalcin* gene in MG-63 cells grown in different concentrations of glucose containing medium for 24 and 96 h periods. Results are means ± SEM of three independent experiments, each in triplicate. *P < 0.05 vs. control (5 mM glucose at 24 h)

Insulin resistance in MG-63 cells was induced first by treating cells with 200 µM palmitate for up to 48 h. Thus, to understand the effect of insulin resistance on *osteocalcin* gene expression, time-course experiments were performed in normal and insulin resistant MG-63 osteoblasts, either untreated or treated with 10 nM insulin for different exposure times (15 min; 6, 24, 48 h). As shown in Fig. 2a, *osteocalcin* mRNA expression was stimulated by insulin, reaching its maximum at 24 h post-treatment and declining thereafter in insulin-sensitive MG-63 cells. In contrast, no variations in *osteocalcin* mRNA expression were observed in palmitate pre-treated, insulin resistant cells, as well as in insulin-unstimulated control cells. These findings, while confirming the role of insulin in the regulation of *osteocalcin* gene expression in this cell line, clearly demostrate that, under conditions of insulin resistance, *osteocalcin* gene expression is precluded.

An inverse correlation between osteocalcin and hyperglycemia has been reported in clinical studies [22, 23]. Therefore, we studied the effects of low (2 mM) and high (25 mM) glucose concentrations on *osteocalcin* gene expression in vitro, in MG-63 cells that were grown in standard and modified glucose containing medium for 24 and 96 h periods. While no significant difference was observed in *osteocalcin* gene expression after the 24-hr incubation period in low and high glucose, a significant increase in *osteocalcin* mRNA levels was observed in cells treated with 2 mM glucose after the 96-h experimental period, together with a reduction of *osteocalcin* mRNA in cells treated with 25 mM glucose during the same 96-h time period (Fig. 2b), recapitulating the situation found in previous clinical studies, in which glycemia inversely correlated with circulating osteocalcin [22, 23].

### Glucose regulates *osteocalcin* gene expression by acting in trans at the transcriptional level

**Fig. 3:**
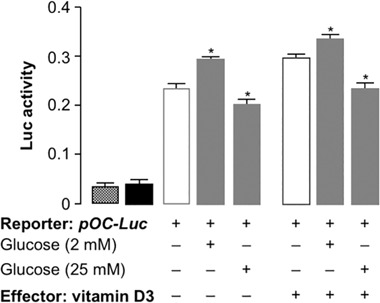
Glucose regulates *osteocalcin* gene transcription. MG-63 cells grown in normal glucose concentration (5 mM), were transfected with pOC-Luc and incubated with low (2 mM) or high (25 mM) glucose concentration, either in the presence or absence of 1 nM 1α,25(OH)2D3, and Luc activity was measured 48 h later. Data are means ± SEM for five separate experiments. Luc activity from the reporter plasmid was normalized by the renilla luc activity produced from control vector (pΔOC-Luc) cotransfected as an internal control. Pattern bar, mock (no DNA); black bar, pΔOC-Luc (without the osteocalcin promoter). *P < 0.05 vs. 5 mM glucose (white bar, in each condition)

Because of the effect of glucose on *osteocalcin* gene expression in MG-63 cells, we then hypothesized that high glucose could be acting, at least in part, by blocking the human *osteocalcin* gene promoter. Methods of bioinformatic analysis were applied to scan the entire *osteocalcin* gene promoter [24], in order to identify putative sites for glucose response. A site at 404 bp before the ATG starting codon was identified, whose sequence, *CACGgggctgacagtag*, was recognized by the carbohydrate response element binding protein, ChREBP, thereby suggesting a possible molecular link between glucose and the *osteocalcin* gene. Therefore, gene reporter experiments were performed to verify the activity of glucose on the promoter region of the *osteocalcin* gene. To this end, a plasmid containing the human *osteocalcin* promoter (pOC) cloned upstream of the *luciferase* (Luc) reporter gene, pOC-Luc [15], was transfected into MG-63 cells, and Luc activity was measured after 48 h from transfection in cells treated with either 2 or 25 mM glucose. As shown in reporter gene assays, pOC-Luc activity was moderately, but significantly reduced in MG-63 cells treated with 25 mM glucose, as compared to cells treated with 2 mM glucose (Fig. 3). The addition to cultured osteoblasts of 1 nM 1α,25-dihydroxyvitamin D3, a factor known to stimulate the *osteocalcin* gene promoter [25], slightly increased Luc activity at both glucose concentrations, further supporting the notion that a relationship indeed exists between glucose concentration and *osteocalcin* gene transcription. In parallel experiments using the same pOC-Luc construct in human embryonic HEK-293 cells (not shown), Luc activity was not modulated by either glucose concentrations or vitamin D, and this was most likely due to the cell specificity of this promoter.

### Glucose and insulin resistance negatively affect osteoblastic MG-63 cell differentiation while inhibiting *osteocalcin* gene expression

**Fig. 4:**
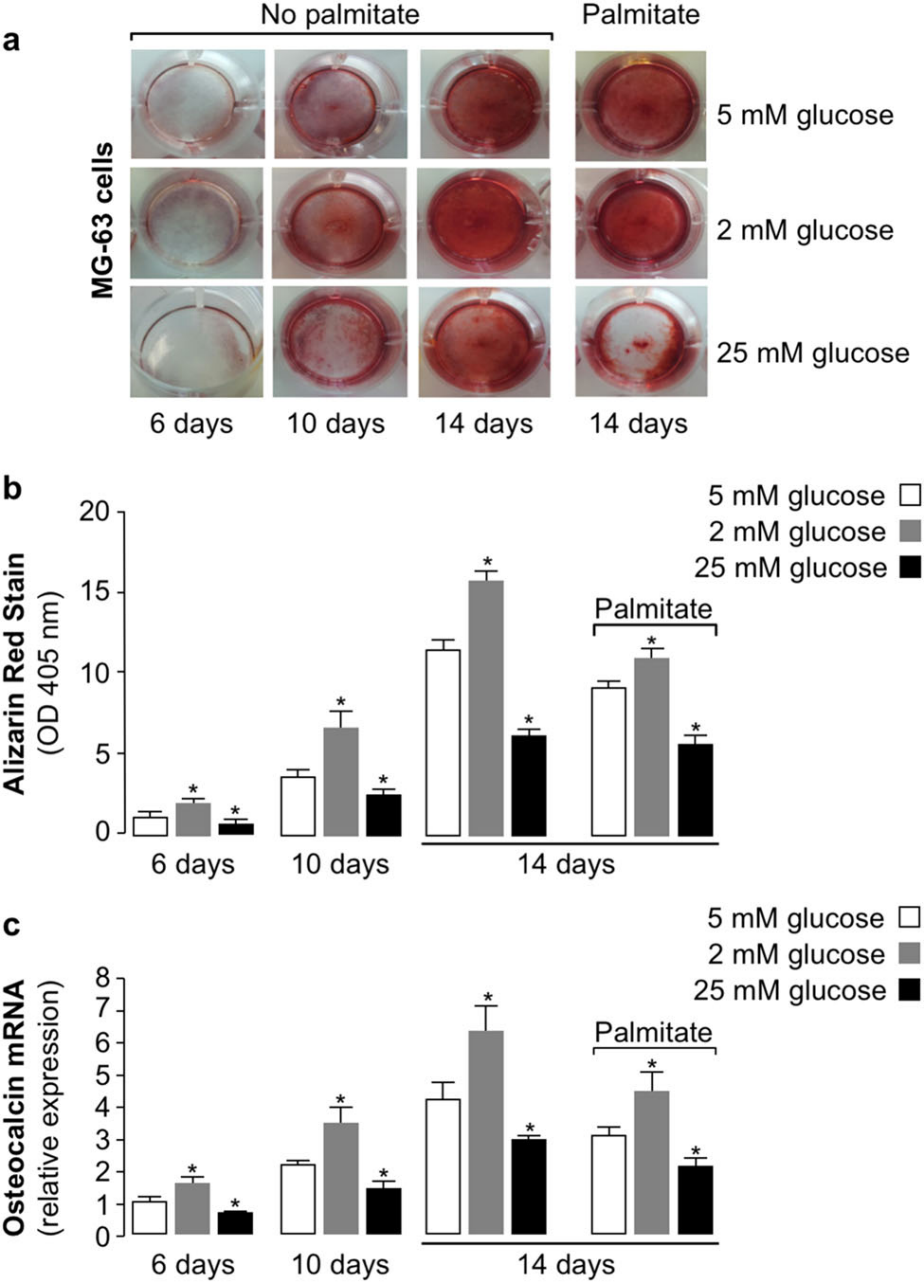
Osteogenic differentiation in MG-63 cells is reduced by glucose and insulin resistance. Alizarin Red S staining (ARS) assay for calcium deposition was carried out at 6, 10, and 14 days in MG-63 cells cultured with osteogenic differentiation medium in the presence of standard (5 mM), low (2 mM), or high (25 mM) glucose concentrations, with or without 200 µM palmitate pretreatment for 48 h. **a** The extent of mineralization nodule formation is shown in each condition after ARS. **b** Semi-quantitative analysis of ARS particles. Extracted solution was measured by the absorbance at 405 nm. Data are from six experiments. *P < 0.05 vs. control (5 mM glucose, in each condition). **c** Results from qRT-PCR of the osteocalcin mRNA in MG-63 cells during osteogenic cell differentiation. Cells were grown in osteogenic differentiation medium in the presence of standard (5 mM), low (2 mM), or high (25 mM) glucose concentration for 6–14 days. The effect of palmitate-induced insulin resistance on osteocalcin mRNA is shown in cells during the 14-days of culture only. Data are means ± SEM for six experiments. *P < 0.05 vs. control (white bar, in each group)

To verify whether glucose could influence osteoblastic differentiation, ARS staining assays [17] were performed in MG-63 cells. This assay measures the osteogenic differentiation in terms of colorimetric intensity. Briefly, during the osteogenic differentiation of osteoblasts, Ca^2+^ ions are deposited in the extracellular matrix, where they subsequently crystallize. Greater is the osteogenic differentiation, more abundant are the calcium crystals in the matrix that could be bound by dye molecules. Treatment of MG-63 cells with 2, 5 and 25 mM glucose were performed at different exposure times up to 14 days. Also, to verify the influence of insulin resistance in osteogenic differentiation, pre-treatment of cells with 200 µM palmitate was performed in parallel experiments, under the same reaction conditions. As shown in Figs. 4a, b, in insulin-sensitive cells, osteogenic differentiation was time-dependent and inversely affected by glucose concentration. In fact, while 2 mM glucose increased MG-63 cell differentiation when compared with 5 mM standard glucose concentration, 25 mM glucose reduced cell differentiation, and this occurred at each time point tested. In experiments with palmitate-induced insulin resistant cells, osteogenic differentiation was considerably reduced with respect to insulin-sensitive cells, and this reduction was observed at each glucose concentration.

To see whether glucose or insulin resistance played a role in osteocalcin expression in MG-63 cells during osteogenic cell differentiation, we performed time-course experiments in which MG-63 cells, either untreated or pre-treated with palmitate to induce in vitro insulin resistance, were incubated with low (2 mM) or high (25 mM) glucose for 6–14 days. As shown in Fig. 4c, MG-63 osteoblasts progressively increased their expression of osteocalcin mRNA during osteogenic differentiation of cells grown in 5 mM standard glucose concentration. Instead, higher and lower amounts of osteocalcin mRNA were detected in MG-63 cells exposed to low and high glucose concentration, respectively, and this is consistent with the above data indicating that osteocalcin expression inversely correlates with glucose concentration in MG-63 cultured cells. Once again, in palmitate-treated insulin resistant cells, osteocalcin mRNA levels were lower, as compared to untreated insulin sensitive MG-63 cells, after the same 14-days differentiation period, thereby indicating that both high glucose and insulin resistance negatively affect ostecalcin expression during osteogenic differentiation.

### Conditioned medium from differentiated MG-63 cells negatively affects *insulin* gene expression and secretion in INS-1 cells

**Fig. 5:**
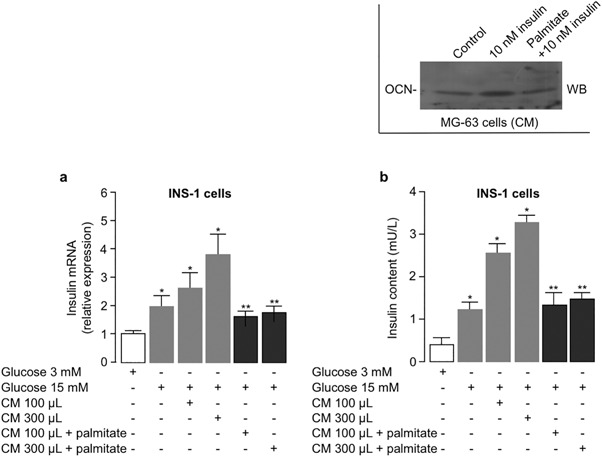
*Insulin* expression and secretion are affected by conditioned medium (CM) from differentiated MG-63 cells. **a** INS-1 cells were incubated with normal culture medium (3 mM glucose, control) or medium containing 15 mM glucose. 100 or 300 µL of CM from insulin sensitive or insulin resistant (200 µM palmitate) MG-63 cells were added to INS-1 cultured cells for 2 h, and qRT-PCR of the insulin cDNA obtained from INS-1 cells in each indicated condition was performed. **b** A rat insulin ELISA kit was used to quantify insulin secretion into the culture medium of INS-1 cells treated as in **a**. Data in **a** and **b** are means ± SEM for six experiments. *P < 0.05 vs. control (white bar, in each condition; **P < 0.05 vs. CM 100 µL and 300 µL, respectively). A representative WB of osteocalcin (OCN) protein abundance in CM from insulin sensitive and insulin resistant MG-63 cells is shown in the inset

To better understand whether a loop existed between bone and pancreatic β-cells, we next treated rat INS-1 cells, a cell line that exhibits glucose-stimulated insulin secretion, with conditioned medium from differentiated MG-63 cells, either untreated or pre-treated with palmitate, in the absence or presence of insulin. Under these conditions, osteocalcin protein abundance increased in conditioned medium from insulin stimulated cells but not in cells pre-treated with palmitate (Fig. 5, inset). Treatment of INS-1 cells with increasing amounts (100 and 300 µL) of conditioned medium from differentiated insulin-sensitive MG-63 cells showed a dose-dependent increase in the levels of insulin mRNA, as compared with glucose-alone treated cells, whereas no rise on insulin mRNA expression was observed in INS-1 cells treated with increasing amounts of conditioned medium from palmitate-treated, insulin-resistant MG-63 cells (Fig. 5a). Additional experiments were carried out to investigate whether, coherently with *insulin* gene expression, conditioned medium from MG-63 cells could also affect insulin secretion in INS-1 cells. To this end, quantitative measurements of insulin in the culture medium of INS-1 cells were achieved by ELISA. As shown in Fig. 5b, the results obtained from these assays paralleled those of the expression studies previously mentioned, reinforcing the concept that insulin sensitivity/insulin resistance in bone cells can influence β-cell insulin production and secretion.

Although we cannot exclude the influence of residual lipotoxicity of palmitate on the impairment of glucose-stimulated insulin production, these results are compatible with the view that osteocalcin may have a direct role in *insulin* gene expression and this effect is negatively affected by resistance of differentiated MG-63 cells to insulin.

### Serum osteocalcin levels inversely correlate with HbA1c

To validate our in vitro findings in a clinical setting, we finally performed a study in humans to see whether a correlation existed between chronic hyperglycemia and/or insulin resistance, and circulating osteocalcin levels. Initially, by employing the non-parametric Mann–Whitney test, we observed that serum osteocalcin levels were markedly lower in diabetic patients (14.7 ng/mL, 12.6–18.3) than in control subjects (20.6 ng/mL, 16.9–24.9) (*P* < 0.001). Similarly, when we considered the glycemic cut-off value of 100 mg/dL, serum osteocalcin was lower in subjects who had a blood glucose value ≥ 100 mg/dL (16.8 ng/mL, 13.9–21.2), and higher in subjects with blood glucose ≤ 100 mg/dL (20.5 ng/mL, 17.5–24.1) (*P = *0.045). To better understand the association of osteocalcin with glyco-metabolic parameters, we performed multiple linear regression analyses, including age, sex, and BMI as covariates. In detail, when the entire enrolled population was considered, serum osteocalcin was negatively associated with HbA1c levels (*P* = 0.001), while no association was observed with neither fasting plasma glucose (*P* = 0.089) or HOMA-IR (*P* = 0.251) (Table 2). Association of osteocalcin with HbA1c was confirmed in a same multiple linear regression analysis including only the control population (Table 2), whereas, no association was detected when only patients with DM2 were considered (Table 2). Also, no difference was observed when the enrolled population was distinguished on the basis of either HOMA-IR < 1.6 (*P* = 0.114) or HOMA-IR > 2.5 (*P* = 0.279). Therefore, altogether, these findings indicate that serum osteocalcin and glucose levels are inversely correlated, while no correlation appears between insulin resistance and osteocalcin.

**Table 2:** Relationship between osteocalcin levels and glucose parameters in enrolled population, control population, and DM2 population

	B	beta	*t*	*P*-value
*Enrolled population*				
FPG	−0.043	−0.236	−1732	0.089
HbA1c	−2.318	−0.432	−3.390	0.001
HOMA-IR	−0.137	−0.153	−1.161	0.251
*Control population*				
FPG	0.096	0.166	0.984	0.332
HbA1c	−7.680	−0.338	−2.188	0.035
HOMA-IR	−0.003	−0.001	−1.161	0.994
*DM2 population*				
FPG	−0.021	−0.158	−0.663	0.517
HbA1c	−4.874	−0.134	−0.622	0.543
HOMA-IR	−0.018	−0.037	−1.161	0.866

Linear regression analysis was used for assessing the effect of the osteocalcin on fasting plasma glucose (FPG), HbA1c, and HOMA-IR, adding age, gender, and BMI as covariates

## Discussion

Until recently, bone has been considered solely as a structural tissue, without metabolic significance. The hypothesis of a link between bone and energy metabolism has been first based on the observation that obesity protects from osteoporosis [26], thus suggesting a common hormonal regulation of bone mass and energy metabolism. Testing this hypothesis allowed the identification of leptin, an adipokine produced by adipose tissue, as a point of convergence between bone and energy metabolism [27]. Additional investigation on this topic led authors to discover that the bone-specific hormone, osteocalcin, while playing important roles in bone remodeling, also contributes to glucose metabolism by affecting both insulin secretion and insulin sensitivity [28]. Furthermore, the link between bone and glucose metabolism is supported by clinical observations indicating that patients with diabetes show an increased risk of fractures because of osteopenia or osteoporosis [29–32]. Similar observations also resulted from studies with animal models [33, 34].

However, although the above-mentioned information, up to now, few in vitro models have been developed to better understand, at the molecular level, how insulin and glucose can regulate osteocalcin. In this study, we used MG-63 human osteoblast-like cells for studying *osteocalcin* gene expression, osteogenic differentiation, and osteblast-mediated insulin secretion. We first set up an in vitro model of insulin-resistant osteoblasts by treating MG-63 cells with palmitate, a typical saturated free fatty acid. We demonstrated in vitro, in MG-63 cells, that insulin resistance, as well as high glucose concentration (25 mM) in the culture medium, inhibited *osteocalcin* gene expression. The human *osteocalcin* promoter has been previously studied [15, 25] and its responsiveness to several agents, including glucocorticoids, vitamin D, and cytokines, such as TGF-β, as well as transcription factors has been reported [25, 35–37]. Using reporter gene analysis, we have shown, for the first time, that glucose decreased *osteocalcin* gene expression at the level of gene transcription, and this may be due to the glucose-mediated effects on the putative ChREBP consensus site in the *osteocalcin* promoter region, as evidenced by bioinformatic analysis.

Based on the assumption that a physiological interaction occurs among bone and pancreas, in relation to the regulation of energy metabolism, and that a positive loop exists between osteocalcin and insulin, co-culture experiments in which osteocalcin-producing COS cells were able to induce insulin secretion from pancreatic islets, but not other hormones, had proved the endocrine activity of osteocalcin in vitro [28]. This aspect was clarified by cell biology experiments, in which co-cultures of pancreatic islets and wild-type osteoblasts stimulated insulin secretion, whereas *osteocalcin-/-* osteoblasts did not [28]. In our study, conditioned medium from insulin sensitive and insulin resistant MG-63 osteoblasts differently affected *insulin* gene expression and secretion in INS-1 cultured β-cells. Although these findings do not prove that differences in insulin secretion unequivocally reflect differences in the release of osteocalcin from insulin-sensitive vs. insulin-resistant osteoblasts, they do provide additional support for a functional relationship between osteocalcin and insulin, with the understanding that we cannot exclude that other MG-63 secreted biomolecules may play a role in this endocrine activity.

Insulin resistance and chronic hyperglycemia are the main metabolic perturbations in patients with type 2 diabetes. In these patients, clinical evidence exists that bone disorders as osteoporosis and bone fractures are more frequent than in other individuals [29–32]. It has been postulated that the reduced levels of osteocalcin may have a role in this context [22, 23]. Results in animal models have encouraged a series of clinical studies, which tried to associate circulating osteocalcin levels with clinical and/or biochemical parameters in different cohorts of patients [38–42]. Although many findings indicate that an inverse correlation exists between total or undercarboxylated osteocalcin and glucose levels, the debate is still open. Since our findings show that insulin resistance and high glucose reduce osteocalcin expression in vitro, we investigated how insulin resistance and/or chronic hyperglycemia affected serum osteocalcin levels in patients. As we found a significant correlation between poor glycemic control and reduced levels of serum osteocalcin, it appears plausible to assume that chronic hyperglycemia (rather than insulin resistance) might have a preminent role in this reduction. Our observation, in this context, well supports previous studies, in which the association of hyperglycemia with low serum osteocalcin levels has been reported [22, 23, 43]. As the number of patients included in our study was relatively low, the significance of this finding deserves further investigation in a larger number of patients. In this regard, the lack of correlation between insulin resistance and osteocalcin levels in our data set may depend on the small sample size of the study, since a correlation between insulin resistance and osteocalcin in patients with insulin resistant syndromes has been observed only in larger cohort studies [44–47]. Thus, future investigation is necessary to establish whether osteocalcin may represent a novel therapeutic target in conditions that range from osteopenia or osteoporosis to insulin resistance and overt diabetes.

## References

[1] Saltiel AR, Kahn CR (2001). Insulin signalling and the regulation of glucose and lipid metabolism. Nature..

[2] Ferron M, Wei J, Yoshizawa T, Del Fattore A, DePinho RA, Teti A, Ducy P, Karsenty G (2010). Insulin signaling in osteoblasts integrates bone remodeling and energy metabolism. Cell.

[3] Harada S, Rodan G (2003). Control of osteoblast function and regulation of bone mass. Nature..

[4] Faienza Maria Felicia, Luce Vincenza, Ventura Annamaria, Colaianni Graziana, Colucci Silvia, Cavallo Luciano, Grano Maria, Brunetti Giacomina (2015). Skeleton and Glucose Metabolism: A Bone-Pancreas Loop. International Journal of Endocrinology.

[5] Ng KW, Martin TJ (2009). New functions for old hormones: Bone as an endocrine organ. Mol. Cell. Endocrinol..

[6] Lombardi G, Perego S, Luzi L, Banfi G (2015). A four-season molecule: osteocalcin. Updates in its physiological roles. Endocrine.

[7] Murshed M, Schinke T, McKee MD, Karsenty G (2004). Extracellular matrix mineralization is regulated locally; different roles of two gla-containing proteins. J. Cell. Biol..

[8] Ducy P, Desbois C, Boyce B, Pinero G, Story B, Dunstan C, Smith E, Bonadio J, Goldstein S, Gundberg C, Bradley A, Karsenty G (1996). Increased bone formation in osteocalcin-deficient mice. Nature..

[9] Mizokami A, Kawakubo-Yasukochi T, Hirata M (2017). Osteocalcin and its endocrine functions. Biochem. Pharmacol..

[10] Kanazawa I (2015). Osteocalcin as a hormone regulating glucose metabolism. World J. Diabetes.

[11] Wei J, Ferron M, Clarke CJ, Hannun YA, Jiang H, Blaner WS, Karsenty G (2014). Bone-specific insulin resistance disrupts whole-body glucose homeostasis via decreased osteocalcin activation. J. Clin. Invest..

[12] Rubin MR (2015). Bone cells and bone turnover in diabetes mellitus. Curr. Osteoporos. Rep..

[13] Arcidiacono B, Iiritano S, Chiefari E, Brunetti FS, Gu G, Foti DP, Brunetti A (2015). Cooperation between HMGA1, PDX-1, and MafA is essential for glucose-induced insulin transcription in pancreatic beta cells. Front. Endocrinol..

[14] Foti D, Chiefari E, Fedele M, Iuliano R, Brunetti L, Paonessa F, Manfioletti G, Barbetti F, Brunetti A, Croce CM, Fusco A, Brunetti A (2005). Lack of the architectural factor HMGA1 causes insulin resistance and diabetes in humans and mice. Nat. Med..

[15] Nakanishi T, Kokubun K, Oda H, Aoki M, Soma A, Taniguchi M, Kazuki Y, Oshimura M, Sato K (2012). Bioluminescence imaging of bone formation using hairless osteocalcin-luciferase transgenic mice. Bone.

[16] Iiritano S, Chiefari E, Ventura V, Arcidiacono B, Possidente K, Nocera A, Nevolo MT, Fedele M, Greco A, Greco M, Brunetti G, Fusco A, Foti D, Brunetti A (2012). The HMGA1-IGF-I/IGFBP system: a novel pathway for modulating glucose uptake. Mol. Endocrinol..

[17] Gregory CA, Gunn WG, Peister A, Prockop DJ (2004). An alizarin red-based assay of mineralization by adherent cells in culture: comparison with cetylpyridinium chloride extraction. Anal. Biochem..

[18] Costa V, Foti D, Paonessa F, Chiefari E, Palaia L, Brunetti G, Gulletta E, Fusco A, Brunetti A (2008). The insulin receptor: a new anticancer target for peroxisome proliferator-activated receptor-γ (PPARγ) and thiazolidinedione-PPARγ agonists. Endocr. Relat. Cancer..

[19] Bianconcini A, Lupo A, Capone S, Quadro L, Monti M, Zurlo D, Fucci A, Sabatino L, Brunetti A, Chiefari E, Gottesman ME, Blaner WS, Colantuoni V (2009). Transcriptional activity of the murine retinol-binding protein gene is regulated by a multiprotein complex containing HMGA1, p54nrb/NonO, protein-associated splicing factor (PSF) and steroidogenic factor 1 (SF1)/liver receptor homologue 1 (LRH-1). Int. J. Biochem. Cell. Biol..

[20] Pullinger CR, Goldfine ID, Tanyolac S, Movsesyan I, Faynboym M, Durlach V, Chiefari E, Foti DP, Frost PH, Malloy MJ, Brunetti A, Kane JP (2014). Evidence that an HMGA1 gene variant associates with type 2 diabetes, body mass index, and high-density lipoprotein cholesterol in a Hispanic-American population. Metab. Syndr. Relat. Disord..

[21] Cifuentes M, García MA, Arrabal PM, Martinez F, Yaňez MJ, Jara N, Weil B, Domínguez D, Medina RA, Nualart F (2011). Insulin regulates GLUT1-mediated glucose transport in MG-63 human osteosarcoma cells. J. Cell. Physiol..

[22] Sarkar PD, Choudhury AB (2013). Relationships between serum osteocalcin levels versus blood glucose, insulin resistance and markers of systemic inflammation in central Indian type 2 diabetic patients. Eur. Rev. Med. Pharmacol. Sci..

[23] Hwang YC, Jeong IK, Ahn KJ, Chung HY (2012). Circulating osteocalcin level is associated with improved glucose tolerance, insulin secretion and sensitivity independent of the plasma adiponectin level. Osteoporos. Int..

[24] Messineo S, Laria AE, Arcidiacono B, Chiefari E, Luque Huertas RM, Foti DP, Brunetti A (2016). Cooperation between HMGA1 and HIF-1 contributes to hypoxia-induced VEGF and visfatin gene expression in 3T3-L1 adipocytes. Front. Endocrinol..

[25] Nakanishi T, Saito R, Taniguchi M, Oda H, Soma A, Yasunaga M, Yamane M, Sato K (2013). In Vivo determination of vitamin D function using transgenic mice carrying a human osteocalcin luciferase reporter gene. Biomed. Res. Int..

[26] Ducy P, Amling M, Takeda S, Priemel M, Schilling AF, Beil FT, Shen J, Vinson C, Rueger JM, Karsenty G (2000). Leptin inhibits bone formation through a hypothalamic relay: a central control of bone mass. Cell.

[27] de Paula FJA, Rosen CJ (2013). Bone remodeling and energy metabolism: new perpectives. Bone Res..

[28] Zoch ML, Clemens TL, Riddle RC (2016). New insights into the biology of osteocalcin. Bone.

[29] Janghorbani M, Feskanich D, Willett WC, Hu F (2006). Prospective study of diabetes and risk of hip fracture: The nurses’ health study. Diabetes Care.

[30] Nicodemus KK, Folsom AR (2001). Type 1 and type 2 diabetes and Incident hip fractures in postmenopausal women. Diabetes Care.

[31] Starup-Linde J, Frost M, Vestergaard P, Abrahamsen B (2017). Epidemiology of fractures in diabetes. Calcif. Tissue Int..

[32] Kemink SAG, Hermus ARMM, Swinkels LMJW, Lutterman JA, Smals AGH (2000). Osteopenia in insulin-dependent diabetes mellitus: prevalence and aspects of pathophysiology. J. Endocrinol. Invest..

[33] Verhaeghe J, Visser WJ, Einhorn TA, Bouillon R (1990). Osteoporosis and diabetes: lessons from the diabetic BB rat. Horm. Res. Paediatr..

[34] Goodman WG, Hori MT (1984). Diminished bone formation in experimental diabetes. Relationship to osteoid maturation and mineralization. Diabetes.

[35] Sims NA, White CP, Sunn KL, Thomas GP, Drummond ML, Morrison NA, Eisman JA, Gardiner EM (1997). Human and murine osteocalcin gene expression: conserved tissue restricted expression and divergent responses to 1,25-dihydroxyvitamin D3 in vivo. Mol. Endocrinol..

[36] Piscopo DM, Johansen EB, Derynck R (2009). Identification of the GATA factor TRPS1 as a repressor of the osteocalcin promoter. J. Biol. Chem..

[37] Chen H, Hays E, Liboon J, Neely C, Kolman K, Chandar N (2011). Osteocalcin gene expression is regulated by wild-type p53. Calcif. Tissue Int..

[38] Hwang YC, Jeong IK, Ahn KJ, Chung HY (2009). The uncarboxylated form of osteocalcin is associated with improved glucose tolerance and enhanced beta-cell function in middle-aged male subjects. Diabetes Metab. Res. Rev..

[39] Strapazzon G, De Toni L, Foresta C (2011). Serum undercarboxylated osteocalcin was inversely associated with plasma glucose level and fat mass in type 2 diabetes mellitus. Osteoporos. Int..

[40] Kindblom JM, Ohlsson C, Ljunggren O, Karlsson MK, Tivesten A, Smith U, Mellström D (2009). Plasma osteocalcin is inversely related to fat mass and plasma glucose in elderly Swedish men. J. Bone Miner. Res..

[41] Kanazawa I, Yamaguchi T, Yamamoto M, Yamauchi M, Kurioka S, Yano S, Sugimoto T (2009). Serum osteocalcin level is associated with glucose metabolism and atherosclerosis parameters in type 2 diabetes mellitus. J. Clin. Endocrinol. Metab..

[42] Neumann T, Lodes S, Kästner B, Franke S, Klehntopf M, Lehmann T, Müllere UA, Wolf G, Sämann A (2016). Osteocalcin, adipokines and their associations with glucose metabolism in type 1 diabetes. Bone.

[43] Maddaloni E, D’Onofrio L, Lauria A, Maurizi AR, Strollo R, Palermo A, Napoli N, Angeletti S, Pozzilli P, Manfrini S (2014). Osteocalcin levels are inversely associated with Hba1c and BMI in adult subjects with longstanding type 1 diabetes. J. Endocrinol. Invest..

[44] Iki M, Tamaki J, Fujita Y, Kouda K, Yura A, Kadowaki E, Sato Y, Moon JS, Tomioka K, Okamoto N, Kurumatani N (2012). Serum undercarboxylated osteocalcin levels are inversely associated with glycemic status and insulin resistance in an elderly Japanese male population: Fujiwara-kyo osteoporosis risk in men (FORMEN) study. Osteoporos. Int..

[45] Díaz-López A, Bulló M, Juanola-Falgarona M, Martinez-González MA, Estruch R, Covas MI, Arós F, Salas-Salvadó J (2013). Reduced serum concentrations of carboxylated and undercarboxylated osteocalcin are associated with risk of developing type 2 diabetes mellitus in a high cardiovascular risk population: a nested case-control study. J. Clin. Endocrinol. Metab..

[46] Schwetz V, Lerchbaum E, Schweighofer N, Hacker N, Trummer O, Borel O, Pieber TR, Chapurlat R, Obermayer-Pietsch B (2014). Osteocalcin levels on oral glucose load in women being investigated for polycystic ovary syndrome. Endocr. Pract..

[47] Garanty-Bogacka B, Syrenicz M, Rać M, Krupa B, Czaja-Bulsa G, Walczak M, Sowiňska-Przepiera E, Syrenicz A (2013). Association between serum osteocalcin, adiposity and metabolic risk in obese children and adolescents. Endokrynol. Pol..

