# Correction to: Insulin and osteocalcin: further evidence for a mutual cross-talk

**DOI:** 10.1007/s12020-017-1480-5

**Published:** 2017-12-23

**Authors:** Francesco L. Bilotta, Biagio Arcidiacono, Sebastiano Messineo, Marta Greco, Eusebio Chiefari, Domenico Britti, Tomoko Nakanishi, Daniela P. Foti, Antonio Brunetti

**Affiliations:** 1 Department of Health Sciences, University “Magna Græcia” of Catanzaro, Viale Europa (Località Germaneto), 88100 Catanzaro, Italy; 2 Laboratory of Molecular Genetics, The Institute of Medical Science, University of Tokyo, 108-8639 Tokyo, Japan

**Correction to: Endocrine** 10.1007/s12020-017-1396-0

The article Insulin and osteocalcin: further evidence for a mutual cross-talk, written by Francesco L. Bilotta, Biagio Arcidiacono, Sebastiano Messineo, Marta Greco, Eusebio Chiefari, Domenico Britti, Tomoko Nakanishi, Daniela P. Foti, Antonio Brunetti, was originally published electronically on the publisher’s internet portal (currently SpringerLink) on 2 September 2017 without open access.

With the author(s)’ decision to opt for Open Choice the copyright of the article changed on 16 November 2017 to © The Author(s) 2017 and the article is forthwith distributed under the terms of the Creative Commons Attribution 4.0 International License (http://creativecommons.org/licenses/by/4.0/), which permits use, duplication, adaptation, distribution and reproduction in any medium or format, as long as you give appropriate credit to the original author(s) and the source, provide a link to the Creative Commons license and indicate if changes were made.

The original article has been corrected.

